# Association between pregnancy and pregnancy loss with COPD in Chinese women: The China Kadoorie Biobank study

**DOI:** 10.3389/fpubh.2022.990057

**Published:** 2022-10-31

**Authors:** Sha Huang, Jia Yi Hee, Yuxun Oswald Zhang, Ruofan Gongye, Siyu Zou, Kun Tang

**Affiliations:** ^1^Vanke School of Public Health, Tsinghua University, Beijing, China; ^2^Department of Maternal and Child Health, Gillings School of Public Health, University of North Carolina, Chapel Hill, NC, United States

**Keywords:** pregnancy, pregnancy loss, spontaneous abortion, induced abortion, stillbirth, COPD

## Abstract

**Background:**

Chronic obstructive pulmonary disease (COPD) is an inflammatory lung disease characterized by airflow blockage. Pregnancy and pregnancy loss may be related to an elevated risk of COPD, although studies have yet to report on this association. Hence, this study aims to investigate the association between pregnancy and pregnancy loss with the risk of COPD among Chinese women.

**Methods:**

Data on 302,510 female participants from the China Kadoorie Biobank were utilized for this study. Multivariable logistic regression, stratified by sociodemographic and lifestyle factors, was employed to obtain the odds ratio (ORs) and 95% confidence intervals (CIs) for the association between pregnancy and pregnancy loss with COPD.

**Results:**

Pregnancy loss was significantly associated with increased risk of COPD (OR 1.19, 95% CI 1.13–1.25), specifically, spontaneous (OR 1.19, 95% CI 1.11–1.29) and induced abortion (OR 1.18, 95% CI 1.12–1.25). Stillbirth, however, was not significantly associated with the risk of COPD (OR 1.09, 95% CI 0.99–1.20). Increasing number of pregnancy losses was associated with increasing risk of COPD (one pregnancy loss: OR 1.14, 95% CI 1.07–1.21, two or more pregnancy loss: OR 1.25, 95% CI 1.17–1.32, and each additional pregnancy loss: OR 1.06, 95% CI 1.03–1.09). A single pregnancy was significantly associated with reduced risk of COPD (OR 0.75, 95% CI 0.59–0.97), although each additional pregnancy was significantly associated with increased risk of COPD (OR 1.03, 95% CI 1.01–1.04).

**Conclusion:**

Pregnancy loss, in particular, spontaneous and induced abortions are associated with increased risk of COPD among Chinese women. A single pregnancy, however, demonstrated protective effects.

## Introduction

Chronic obstructive pulmonary disease (COPD) is a chronic and progressive inflammatory lung disease characterized by airflow blockage. Emphysema and chronic bronchitis, largely caused by cigarette smoking, are common contributors to COPD (1). In 2019, COPD was ranked by the World Health Organization (WHO) as the third leading cause of death worldwide (2). According to 2018 statistics, an estimated 8.6% of the population in China have been diagnosed with COPD (3). The global aging population contributes in part to the rising prevalence of COPD-related deaths. The use of solid fuel for cooking, smoking, adiposity, and genetics have also been identified as risk factors for COPD (4, 5). However, while cigarette smoking is a known major contributor to COPD, 20.5% of individuals with COPD are non-smokers (1).

Factors associated with reproduction unique to women have also been hypothesized to contribute to COPD. Studies have detected an elevated expression of the pregnancy-associated plasma protein-A (PAPP-A), a placental protein present in abundance in the blood circulation of pregnant women, as well as in patients with asthma, lung cancer, and COPD (6–9). Studies have also demonstrated that pregnancy loss is associated with an increased risk of chronic diseases, such as diabetes, stroke, and atherosclerotic disease (10–14). The presence of inflammatory markers due to these diseases may contribute to the increase in risk of COPD (15, 16).

Pregnancy loss is a common adverse pregnancy outcome; an estimated 2 million pregnancies result in stillbirths, 23 million pregnancies result in spontaneous abortions (17, 18), while a further 73 million pregnancies are terminated worldwide annually (19). In 1983, during the one-child policy in China, there was an estimated 14.4 million abortions; the rate of abortions peaked at 56.07% in the 1980s. There was a gradual downward tendency in the abortion rate from 44.95 to 19.30% in 1991 to 2000 (20). Given that pregnancy loss was a common outcome of pregnancy during the one-child policy, and because pregnancy and pregnancy loss have been hypothesized to contribute to increased risk of COPD, the aim of this study is to investigate the association between previous occurrences of pregnancy and pregnancy loss (including spontaneous abortion, induced abortion, and stillbirth) with the risk of COPD using data from the China Kadoorie Biobank. To our knowledge, no study has assessed the association between pregnancy and pregnancy loss with the risk of COPD.

## Materials and methods

### Study population

Data from the China Kadoorie Biobank (CKB), a large prospective database established by the Chinese Center for Disease Control and Prevention and the University of Oxford was utilized for this study. Detailed information about the study design and procedures of CKB have been previously reported elsewhere (21, 22). Briefly, 512,715 participants aged between 30 and 79 years old from 10 urban and rural areas in China, were recruited between September 2004 to May 2008. From an initial cohort of 512,715 participants, 210,205 participants were excluded as they were males. The remaining 302,510 participants were included in the final analyses.

### Assessment of exposure, covariates, and outcome

At the local study assessment centers, trained health workers administered an electronic questionnaire that collected sociodemographic information, such as region of residence, educational level, occupation, household income, healthcare cover, and medical history, as well as lifestyle factors such as diet, alcohol consumption, and smoking status of participants. The number of live births, spontaneous abortions, induced abortions, and stillbirths were collected for female participants. Physical measurements, such as standing height and weight, were measured by trained staff using standard instruments and protocols. COPD diagnoses was ascertained *via* medical records and electronic linkage to the national health insurance (HI) system (21, 22). However, self-reported COPD diagnoses were used in a small percentage (2.0%) of participants who were not linked to the HI system (5).

### Statistical analysis

Baseline characteristics were presented as mean (standard deviation) for continuous variables and percentages for categorical variables. Missing values were treated and reported as missing in all analyses. Pregnancy and pregnancy losses were re-categorized into 0, 1, and 2 or more. Logistic regression was utilized to obtain the odds ratio (OR) and 95% confidence intervals (95% CI) for the association between pregnancy and pregnancy loss, including spontaneous abortion, induced abortion, and stillbirth, with the risk of COPD. All analyses were adjusted for age, region (urban/rural), body mass index (BMI), educational level (elementary school and below/middle and high school/ university and above), annual household income (low/middle/high), metabolic equivalent of task (MET), smoking (never/ever), and alcohol consumption (never/ever). Analyses for pregnancy loss were restricted to parous women and was adjusted for the number of live births, and where appropriate, the number of spontaneous abortions, induced abortions, and stillbirths. Covariates were selected based on prior knowledge and published literature.

Subgroup analyses were performed to obtain the ORs and 95% CIs for the risk of COPD as associated with pregnancy or pregnancy loss by age (30–39.9, 40–49.9, 50–59.9, and ≥60 years), BMI (<25 and ≥25 kg/m^2^), region (rural and urban), educational level (elementary school and below, middle and high school, and university and above), annual income (<5,000, 5,000–19,999, and ≥20,000 yuan/year), MET (<20, 20–29.9, and ≥30 h/day), smoking status (smoker and non-smoker), alcohol consumption (alcohol drinker and non-alcohol drinker), and tuberculosis diagnosis (yes and no). The effect of pregnancy/pregnancy loss and baseline characteristics on the outcome of COPD was assessed using an interaction term. All statistical analyses were performed using Statistics Analysis System (SAS) (version 9.4; SAS Institute Inc., NC, USA).

### Ethics approval

The CKB database has been given ethical approval by the University of Oxford, Peking University, the China National Center for Disease Control and Prevention (CDC), and the institutional review boards at the local CDCs of the 10 study regions. Written informed consent was provided by all participants for participation and to allow for access to their medical records (22).

## Results

### Baseline characteristics of participants

The characteristics of the participants are presented in Table 1 and Supplementary Table 1. Among the 302,510 women included in the analyses, 99.05% (*n* = 299,582) reported having ever been pregnant, of which, 61.52% had a history of pregnancy loss, 9.06% had a history of spontaneous abortion, 52.48% had a history of induced abortion, and 5.69% had a history of stillbirth. Women who reported having ever been pregnant had a mean age of 51.46 years, mean BMI of 23.82 kg/m^2^, mean MET values of 20.44 h/day, and a mean number of livebirths of 2.24. The majority of women resided in rural areas (55.59%), had an education of primary education or below (56.85%), and had household incomes between 5,000 and 19,999 yuan (49.13%). Only 5.05% and 36.37% of these women smoked and drank alcohol, respectively.

**Table 1:** Baseline characteristics of study participants.

**Characteristics**	**Total**	**History of pregnancy**
N	302,510	299,582
Age (years), mean (SD)	51.46 (10.48)	51.46 (10.47)
BMI (kg/m^2^), mean (SD)	23.82 (3.46)	23.82 (3.46)
**Socioeconomic factors**		
Region, %		
Rural	55.43	55.59
Urban	44.57	44.41
**Educational level, %**		
Elementary school and below	56.72	56.85
Middle and high school	38.84	38.80
University and above	4.44	4.35
**Household income, %**		
Low (<5,000)	10.16	10.14
Middle (5,000–19,999)	49.16	49.13
High (≥20,000)	40.69	40.74
**Lifestyle factors**		
Physical activity (MET hours/day), mean (SD)	20.42 (12.76)	20.44 (12.76)
Ever regular smoker, %	5.07	5.05
Ever regular alcohol drinker, %	36.42	36.37
**Medical and reproductive history**		
History of tuberculosis, %	1.13	1.11
History of spontaneous abortion, %	8.98	9.06
History of induced abortion, %	51.98	52.48
History of stillbirth, %	5.63	5.69
Number of live births, mean (SD)	2.24 (1.34)	2.24 (1.34)

BMI, body mass index; MET, metabolic equivalent of tasks.

### Pregnancy and pregnancy loss with the risk of COPD

The associations between pregnancy and pregnancy loss, including spontaneous abortion, induced abortion, and stillbirth, with the risk of COPD are presented in Table 2.

**Table 2:** Adjusted odds ratios (95% confidence intervals) for COPD risk associated with number of pregnancies and pregnancy losses.

	**Number of participants**	**Number of events**	**Model 1, OR (95% CI)**	**Model 2, OR (95% CI)**
**Pregnancies** ^**a**^
*Ever vs. never*			0.82 (0.65, 1.03)	0.84 (0.67, 1.05)
0	2,881	78	1.00 (reference)	1.00 (reference)
1	27,386	400	0.53 (0.42, 0.68)**	0.75 (0.59, 0.97)*
≥2	272,196	6,266	0.85 (0.68, 1.06)	0.85 (0.68, 1.07)
*Peradditional* ^f^			1.16 (1.15, 1.17)**	1.03 (1.01, 1.04)**
**Pregnancy losses** ^**b**^				
*Evervs*.*never*^g^			1.15 (1.09, 1.21)**	1.19 (1.13, 1.25)**
0	115,283	2,352	1.00 (reference)	1.00 (reference)
1	93,409	2,105	1.11 (1.04, 1.18)**	1.14 (1.07, 1.21)**
≥2	90,887	2,209	1.20 (1.13, 1.27)**	1.25 (1.17, 1.32)**
*Peradditional* ^f^			1.06 (1.03, 1.09)**	1.06 (1.03, 1.09)**
**Spontaneous abortion** ^**c**^
*Evervs*.*never*^g^			1.32 (1.22, 1.42)**	1.19 (1.11, 1.29)**
0	272,424	5,896	1.00 (reference)	1.00 (reference)
1	21,412	612	1.33 (1.22, 1.45)**	1.22 (1.12, 1.33)**
≥2	5,744	158	1.28 (1.09, 1.50)**	1.10 (0.93, 1.29)
*Peradditional* ^f^			0.97 (0.88, 1.08)	0.94 (0.84, 1.04)
**Induced abortion** ^**d**^
*Evervs*.*never*^g^			1.04 (0.99, 1.10)	1.18 (1.12, 1.25)**
0	142,348	3,098	1.00 (reference)	1.00 (reference)
1	83,153	1,844	1.02 (0.96, 1.08)	1.13 (1.06, 1.20)**
≥2	74,079	1,724	1.07 (1.01, 1.14)*	1.26 (1.18, 1.34)**
*Peradditional* ^f^			1.04 (1.004, 1.07)*	1.07 (1.04, 1.10)**
**Stillbirth** ^**e**^
*Evervs*.*never*^g^			1.49 (1.36, 1.62)**	1.09 (0.99, 1.20)
0	282,538	6,123	1.00 (reference)	1.00 (reference)
1	13,174	405	1.43 (1.29, 1.59)**	1.11 (1.002, 1.23)*
≥2	3,867	138	1.67 (1.41, 1.98)**	1.03 (0.86, 1.23)
*Peradditional* ^f^			1.07 (0.97, 1.19)	0.93 (0.83, 1.05)

Model 1: Unadjusted.

Model 2: Adjusted for age, region, BMI, level of highest education, annual household income, physical activity, smoking, and alcohol consumption. Model 2 analyses for pregnancy loss, spontaneous abortion, induced abortion, and stillbirth were additionally adjusted for number of live births, and (where appropriate) number of spontaneous abortions, induced abortions, and stillbirths.

*p < 0.05; **p < 0.01.

^a^Missing value = 47.

^b^Missing value = 3.

^c^Missing value = 2.

^d^Missing value = 2.

^e^Missing value = 3.

^f^Analyses are restricted to women with at least one pregnancy, pregnancy loss, spontaneous abortion, induced abortion, or stillbirth, respectively.

^g^Analyses are restricted to women with at least one pregnancy.

There was no statistically significant difference in the risk of COPD between gravid and nulligravid women, OR 0.84 (95% CI 0.67–1.05). However, when stratified by the number of pregnancies, gravid women with a single pregnancy were significantly less likely to be diagnosed with COPD, OR 0.75 (95% CI 0.59–0.97) although the association was not statistically significant for two or more pregnancies, OR 0.85 (95% CI 0.68–1.07). Conversely, each additional pregnancy was significantly associated with increased risk of COPD (OR 1.03, 95% CI 1.01–1.04).

Compared to women without a history of pregnancy loss, women with a history of pregnancy loss were significantly more likely to be diagnosed with COPD, OR 1.19 (95% CI 1.13–1.25). Increasing number of pregnancy losses was significantly associated with increasing risk of COPD; OR 1.14 (95% CI 1.07–1.21) and OR 1.25 (95% CI 1.17–1.32) for one, and two or more pregnancies, respectively. Each additional pregnancy loss was also significantly associated with increased odds of COPD, OR 1.06 (95% CI 1.03–1.09).

The associations were similar directionally for the various types of pregnancy losses; OR 1.19 (95% CI 1.11–1.29), OR 1.18 (95% CI 1.12–1.25), and OR 1.09 (95% CI 0.99–1.20) for a history of spontaneous abortion, induced abortion, and stillbirth, respectively. However, the association between stillbirth and the risk of COPD was not significant. Increasing number of induced abortions was also found to be significantly associated with increasing risk of COPD; OR 1.13 (95% CI 1.06–1.20), and OR 1.26 (95% CI 1.18–1.34) for one, and two or more pregnancy losses, respectively.

### Pregnancy and pregnancy loss with the risk of COPD, stratified by baseline characteristics

The associations between pregnancy and pregnancy loss with the risk of COPD, stratified by age, BMI, region, educational level, household income, MET, smoking status, alcohol consumption, and tuberculosis diagnosis is presented in Table 3. While the associations between spontaneous abortion, induced abortion, and stillbirth with the risk of COPD, stratified by baseline characteristics, were not presented on, the associations can be found in Supplementary Table 2.

**Table 3:** Adjusted odds ratios (95% confidence intervals) for COPD risk associated with number of pregnancies and pregnancy losses, stratified by baseline characteristics.

	**Number of**	**Number of**	**Pregnancy**	***p* for**	**Number of**	**Number of**	**Pregnancy loss**	***p* for**
	**participants**	**events**	**OR (95% CI)**	**interaction**	**participants**	**events**	**OR (95% CI)**	**interaction**
**Age**				0.75				0.12
30–39.9	48,041	444	1.03 (0.46, 2.33)		47,399	307	1.37 (1.11, 1.70)**	
40–49.9	93,549	1,295	0.81 (0.49, 1.36)		92,726	911	1.23 (1.09, 1.40)**	
50–59.9	93,867	2,264	0.90 (0.58, 1.39)		93,118	1,454	1.16 (1.06, 1.27)**	
≥60	67,053	2,663	0.76 (0.55, 1.06)		66,339	1,642	1.16 (1.06, 1.26)**	
**BMI**				0.59				
<25	198,799	4,407	0.80 (0.61, 1.04)		196,774	2,816	1.19 (1.12, 1.27)**	0.82
≥25	103,711	2,259	0.91 (0.59, 1.39)		102,808	1,498	1.18 (1.08, 1.29)**	
**Region**				0.22				
Rural	167,682	3,367	0.75 (0.54, 1.04)		166,530	1,842	1.08 (0.99, 1.17)	0.14
Urban	134,828	3,299	0.90 (0.67, 1.23)		133,052	2,472	1.24 (1.12, 1.37)**	
**Educational level**				0.39				
Elementary school and below	171,580	4,473	0.89 (0.65, 1.21)		170,305	2,644	1.19 (1.10, 1.28)**	0.15
Middle and high school	117,491	1,903	0.86 (0.58, 1.29)		116,249	1,448	1.08 (0.93, 1.25)	
University and above	13,439	290	0.54 (0.30, 0.96)*		13,028	222	0.98 (0.65, 1.48)	
**Household income**				0.20				0.76
<5,000	30,720	1,068	1.41 (0.73, 2.76)		30,367	554	1.19 (1.05, 1.35)**	
5,000–19,999	148,702	2,915	0.76 (0.55, 1.04)		147,173	1,854	1.22 (1.13, 1.32)**	
≥20,000	123,088	2,683	0.76 (0.53, 1.09)		12,204	1,906	1.16 (1.07, 1.27)**	
**MET**				0.63				0.06
<20	177,743	4,415	0.89 (0.68, 1.17)		175,745	2,911	1.17 (1.09, 1.25)**	
20–29.9	58067	1,114	0.81 (0.44, 1.47)		57,617	689	1.13 (0.997, 1.29)	
≥30	66,700	1137	0.65 (0.37, 1.13)		66,220	714	1.24 (1.09, 1.41)**	
**Smoking status**				0.40				0.21
Never smoker	287,180	5,996	0.80 (0.64, 1.02)		284,454	3,909	1.19 (1.11, 1.27)**	
Ever smoker	15,330	670	1.10 (0.53, 2.26)		15,128	405	0.95 (0.77, 1.16)	
**Alcohol consumption**				0.03				0.17
Never drinker	192,323	4,658	1.02 (0.75, 1.38)		190,625	2,945	1.18 (1.09, 1.27)**	
Ever drinker	110,187	2,008	0.62 (0.44, 0.86)**		108,957	1,369	1.08 (0.95, 1.23)	
**Tuberculosis**				0.24				0.36
No	299,087	6,431	0.83 (0.66, 1.04)		296,254	4,154	1.19 (1.13, 1.25)**	
Yes	3,423	235	1.66 (0.60, 4.61)		3,328	160	1.14 (0.84, 1.54)	

Adjusted for age, region, BMI, level of highest education, annual household income, physical activity, smoking, and alcohol consumption. Analyses for pregnancy loss, spontaneous abortion, induced abortion, and stillbirth, were additionally adjusted for number of live births, and (where appropriate) number of spontaneous abortions, induced abortions, and stillbirths. Analyses for pregnancy, pregnancy loss, spontaneous abortion, induced abortion, and stillbirth, respectively, are among women with at least one pregnancy or at least one pregnancy loss, spontaneous abortion, induced abortion, stillbirth BMI, body mass index; MET, metabolic equivalent of tasks. *p < 0.05; **p < 0.01.

Pregnancy was negatively associated with the risk of COPD in all subgroup analyses, except for women between the age 30–39.9, who had household incomes of <5,000 yuan, who smoked, is a non-alcohol drinker, and who has tuberculosis. However, the association between pregnancy and the risk of COPD in women between the ages 30–39.9, 40–49.9, 50–59.9, and above 60, whose BMI was <25 and ≥25, who resided in rural and urban regions, who had elementary school or middle and high school education, who had household incomes of <5,000 yuan, 5,000–19,999 yuan, and ≥20,000 yuan, who had MET values <20, 20–29.9 or ≥30, who smoked or do not smoke, who was a non-alcohol drinker, and who had or did not have tuberculosis were not statistically significant. Of statistical significance was the association between pregnancy and the risk of COPD in women who had educational levels university and above (OR 0.54, 95% CI 0.30–0.96) and who were alcohol drinkers (OR 0.62, 95% CI 0.44–0.86). The interactions between pregnancy and baseline characteristics on the risk of COPD were not statistically significant except for the effect of alcohol consumption on the association between pregnancy and COPD (*p* for interaction = 0.03).

Pregnancy loss was positively associated with the risk of COPD in all subgroup analyses, except in women with educational levels of university and above and in smokers, for which there was an inverse association. However, the association between pregnancy loss and the risk of COPD in women who resided in rural regions, who had educational level of middle school and above, whose MET value was between 20.0 and 29.9, who smoked, who was an alcohol-drinker, and who have tuberculosis were not statistically significant. Of statistical significance was the association between pregnancy loss and the risk of COPD in women between the ages 30–39.9, 40–49.9, 50–59.9, and above 60 (30–39.9: OR 1.37, 95% CI 1.11–1.70; 40–49.9: OR 1.23, 95% CI 1.09–1.40; 50–59.9: OR 1.16, 95% CI 1.06–1.27; and above 60: OR 1.16, 95% 1.06–1.26), whose BMI was <25 and ≥25 (BMI <25: OR 1.19, 95% CI 1.12–1.27; BMI ≥25: OR 1.18, 95% CI 1.08–1.29), who resided in urban regions (OR 1.24, 95% CI 1.12–1.37), with educational levels elementary school and below (OR 1.19, 95% 1.10–1.28), who had household incomes of <5,000 yuan, 5,000–19,999 yuan, and ≥20,000 yuan (<5,000 yuan: OR 1.19, 95% CI 1.05–1.35; 5,000–19,999 yuan: OR 1.22, 95% CI 1.13–1.32; ≥20,000 yuan: OR 1.16, 95% CI 1.07–1.27), who had MET values <20 or ≥30 (MET <20: OR 1.17, 95% CI 1.09–1.25 and MET ≥30 OR 1.24, 95% CI 1.09–1.41), who do not smoke (OR 1.19, 95% CI 1.11–1.27), who was a non-alcohol drinker (OR 1.18, 95% CI 1.09–1.27), and who did not have tuberculosis (OR 1.19, 95% CI 1.13–1.25). No significant interactions were observed between pregnancy loss and baseline characteristics on the risk of COPD (*p* for interaction >0.05).

The association between spontaneous and induced abortion with the risk of COPD in the subgroup analyses was broadly similar to that of pregnancy loss in general, while the association between pregnancy and stillbirth with the risk of COPD in the subgroup analyses was largely non-significant (Supplementary Table 2).

## Discussion

In this cohort of Chinese women, a history of pregnancy loss, specifically, spontaneous, and induced abortion, was associated with increased risk of COPD. A single pregnancy, however, was significantly associated with reduced risk of COPD, although each subsequent additional pregnancy was significantly associated with increased risk of COPD.

At present, to our knowledge, no study has examined the association between pregnancy loss and COPD. However, it is physiologically plausible that pregnancy loss is associated with increased risk of COPD. Recent studies have reported that levels of PAPP-A, a metalloproteinase secreted by the placenta, is elevated in individuals with COPD (9). Elevated PAPP-A levels can significantly influence the reproductive condition and maternal PAPP-A levels tend to increase with both gravidity and parity (23, 24). High concentration of PAPP-A was also demonstrated to be associated with increased number of miscarriages and fetal loss (24, 25). Studies have also reported that women who experienced pregnancy loss were at higher risk for cardiovascular diseases and diabetes (10, 14, 26, 27), which have been associated with reduced lung function (28). Moreover, decreased lung function have been linked to increased levels of markers for inflammation (29), and increasing evidence indicates that chronic inflammatory and immune responses play key roles in the pathogenesis of different forms of lung conditions, including COPD (15, 30). Several inflammatory cells and their mediators, including C-reactive protein (CRP), tumor necrosis factor (TNF)-α, and interleukins (IL-6 and IL-8) have been demonstrated to participate in the inflammatory response in COPD (30, 31).

Our findings demonstrated that a single pregnancy was negatively associated with the risk of COPD. On the contrary, however, each additional pregnancy was positively associated with the risk of COPD. Based on 2016 statistics, the age of women at first birth in China is on average, 26.9 years old (32). Hence, the risk of COPD at the age of first pregnancy is likely to be low. Given that each additional pregnancy is likely to occur with increasing age, and that advancing age contributes to increased risk of COPD, this could in part explain the association between each additional pregnancy with increased risk of COPD. In addition, given the high rates of induced abortions (51.98%) during the period of the one-child policy that coincided with data collection of the present study, increasing number of pregnancy losses, specifically induced abortions, may have resulted in the increased prevalence of COPD, as increased number of pregnancy losses was associated with increased risk of COPD in our study.

Considering that pregnancy causes various physiological changes that may perturb metabolic homeostasis resulting in hormonal imbalance, fat accumulation, decreased insulin sensitivity, and increased insulin resistance (33–36). During pregnancy and pregnancy loss, including spontaneous and induced abortion, anatomic, and physiologic changes may lead to increased maternal susceptibility to both acute and chronic pulmonary diseases, thereby increasing the risk of COPD. In addition, several studies have raised the impact of parity on lung function and provide evidence that multiparity is associated with a tendency toward lung-hyperinflation and lower lung function (37–39). However, due to the lack of relevant studies investigating pregnancy and pregnancy loss on COPD risk, we are unable to understand the specific triggering and regulatory mechanisms of pregnancy, and pregnancy loss on the risk of COPD.

Several unexpected findings were found. We found that pregnancy loss and spontaneous abortion was associated with increased risk of COPD in participants residing in urban areas, but not for participant residing in rural areas. This defies the assumption that because women residing in urban areas enjoy higher levels of medical resources, they should have a better prognosis in cases of pregnancy loss compared to women residing in rural areas. A possible reason to explain the significance of the association between pregnancy loss and spontaneous abortion with the risk of COPD in women residing in urban areas could be the China one-child policy implemented in 1980. The one-child policy was more strictly implemented in urban areas as compared to rural areas, resulting in higher rates of induced abortions. This suggests that higher rates of induced abortions in urban areas are of importance to the association between pregnancy loss and COPD. In addition, we found a lack of association between pregnancy loss and COPD in smokers and alcohol drinkers, a negative association between pregnancy and COPD risk in alcohol drinkers, and a non-significant association between pregnancy with COPD risk in smokers. However, because smokers and alcohol drinkers are known to take a longer time to conceive, and because of the small sample size of this group of women, the association between pregnancy and pregnancy loss with the risk of COPD may be masked.

### Strengths and limitations

Overall, this study has several strengths. To our knowledge, this is the first study to report on the association between pregnancy loss and COPD risk in the Chinese population, and as data collection was conducted in 10 different areas in China, the findings of this study can be better generalized. Furthermore, the survey covered a comprehensive range of demographic, socioeconomic, and lifestyle information, allowing for greater control of confounding. In addition, data was collected using a standardized approach with stringent quality control, ensuring greater reliability and reproducibility.

However, this study also has several limitations. First, pregnancy and pregnancy loss were based on self-reporting, which may result in recall bias. Second, although stratified analysis has been performed to identify associated factors, we were not able to include all the conditions or risk factors along the different phases for both pregnancy loss and comorbidities. Third, residual confounding from other known or unknown risk factors, such as exposure to biofuel, may still exist despite our best efforts to control for a wide range of confounders. Fourth, as the ages at pregnancy and pregnancy loss were not collected, we were unfortunately unable to stratify for age at which pregnancy and pregnancy loss occurred. However, we did stratify for age at survey and observed no significant differences among women in different age groups. Future epidemiological studies are still warranted to verify and better understand the potential influence of age at pregnancy and pregnancy loss on the observed association. Fifth, as spirometry, a common test utilized to diagnose COPD may not have been utilized in the clinical diagnosis of COPD, there may be non-differential misdiagnoses of COPD, resulting in the underestimation of the association. Finally, as the study was a cross-sectional study, a causal relationship between pregnancy and pregnancy loss with COPD cannot be inferred, and further validation is needed.

## Conclusion

Our study demonstrates that pregnancy loss, specifically spontaneous abortion, and induced abortion, is significantly positively associated with the risk of COPD in Chinese women. Additionally, it was observed that increasing number of pregnancy losses was associated with increased risk of COPD. However, given the cross-sectional nature of this study, further prospective cohort studies are needed to validate the findings of this study. Pregnancy, pregnancy loss, and COPD incur a complex cascade with other biological and non-biological factors, necessitating further investigation of the pathological processes associated with pregnancy loss and COPD.

## Data availability statement

The raw data supporting the conclusions of this article will be made available by the authors, without undue reservation.

## Ethics statement

The studies involving human participants were reviewed and approved by the University of Oxford, Peking University, the China National Center for Disease Control and Prevention (CDC), and the Institutional Review Boards at the local CDCs of the 10 regions before the start of the survey. The patients/participants provided their written informed consent to participate in this study.

## Author contributions

SH, YZ, and RG: data analysis and original draft preparation. SH, JH, YZ, SZ, and KT: review and editing. KT: directed and designed the research. All authors have read and agreed to the published version of the manuscript.

## Funding

This work was supported by the National Key Research and Development Program of China (2016YFC0900500, 2016YFC0900501, and 2016YFC0900504), and the Wellcome Trust in the UK (088158/Z/09/Z and 104085/Z/14/Z). The China Kadoorie Biobank baseline survey and the first re-survey was supported by the Kadoorie Charitable Foundation in Hong Kong.

## Conflict of interest

The authors declare that the research was conducted in the absence of any commercial or financial relationships that could be construed as a potential conflict of interest.

## Publisher's note

All claims expressed in this article are solely those of the authors and do not necessarily represent those of their affiliated organizations, or those of the publisher, the editors and the reviewers. Any product that may be evaluated in this article, or claim that may be made by its manufacturer, is not guaranteed or endorsed by the publisher.
