# Supplementary material for: Association between pregnancy and pregnancy loss with COPD in Chinese women: The China Kadoorie Biobank study
Source: Front Public Health. 2022 Oct 31;10:990057. doi: 10.3389/fpubh.2022.990057 (PMC9660272; doi:10.3389/fpubh.2022.990057)
Supplement: Supplementary file 1 [file Data_Sheet_1.docx]

Association between pregnancy and pregnancy loss with COPD in Chinese women: The China Kadoorie Biobank study

Sha Huang^1^, Jiayi Hee^1^, Yuxun Oswald Zhang^1^, Ruofan Gongye^2^, Siyu Zou^1^, Kun Tang^1^*

^1^ Vanke School of Public Health, Tsinghua University, Beijing, China

^2^ Department of Maternal and Child Health, Gillings School of Public Health, University of North Carolina–Chapel Hill, NC, USA

*** Correspondence:**Kun Tang
[tangk@tsinghua.edu.cn](mailto:tangk@tsinghua.edu.cn)

**Table of Contents**

Supplementary Table 1. Baseline characteristics of study participants by number of pregnancies and pregnancy losses

Supplementary Table 2. Adjusted odds ratios (95% confidence intervals) for COPD risk associated with number of pregnancy losses, stratified by baseline characteristics

| Supplementary Table 1. Baseline characteristics of study participants by number of pregnancies and pregnancy losses | | | | | | | |
| --- | --- | --- | --- | --- | --- | --- | --- |
| **Characteristics** | **Number of pregnancy ^a^** | | |  | **Number of pregnancy loss ^b^** | | |
|  | **0** | **1** | **≥2** |  | **0** | **1** | **≥2** |
| N (%) | 2881 (0.95) | 27386 (9.05) | 272196 (89.99) |  | 115283 (38.48) | 93409 (31.18) | 90887 (30.34) |
| **Age** (years), mean (SD) | 50.95 (11.63) | 44.90 (7.86) | 52.12 (10.47) |  | 51.71 (10.73) | 51.30 (10.37) | 51.32 (10.24) |
| **BMI** (kg/m^2^), mean (SD) | 23.44 (3.78) | 23.69 (3.35) | 23.83 (3.47) |  | 23.68 (3.49) | 23.84 (3.44) | 23.97 (3.43) |
| **Socioeconomic factors** |  |  |  |  |  |  |  |
| Region, % |  |  |  |  |  |  |  |
| Rural | 39.08 | 37.29 | 57.43 |  | 68.43 | 49.97 | 45.08 |
| Urban | 60.92 | 62.71 | 42.57 |  | 31.57 | 50.03 | 54.92 |
| Educational level, % |  |  |  |  |  |  |  |
| Elementary school and below | 43.32 | 32.47 | 59.30 |  | 66.66 | 53.39 | 47.96 |
| Middle and high school | 42.52 | 57.93 | 36.88 |  | 30.6 | 41.6 | 46.35 |
| University and above | 14.16 | 9.60 | 3.82 |  | 2.74 | 5.02 | 5.7 |
| Household income, % |  |  |  |  |  |  |  |
| Low (< 5000) | 12.08 | 5.25 | 10.63 |  | 13.88 | 8.02 | 7.57 |
| Middle (5000–19,999) | 52.31 | 43.76 | 49.67 |  | 52.23 | 45.63 | 48.79 |
| High (≥20,000) | 35.61 | 50.99 | 39.71 |  | 33.9 | 46.36 | 43.64 |
| **Lifestyle factors** |  |  |  |  |  |  |  |
| Physical activity (MET hours/day), mean (SD) | 17.91 (11.90) | 22.81 (12.94) | 20.20 (12.72) |  | 21.34 (12.91) | 20.50 (12.96) | 19.24 (12.27) |
| Ever regular smoker, % | 6.56 | 3.45 | 5.21 |  | 4.52 | 4.71 | 6.07 |
| Ever regular alcohol drinker, % | 41.93 | 41.73 | 35.83 |  | 31.47 | 36.77 | 42.18 |
| **Medical and reproductive history** |  |  |  |  |  |  |  |
| History of tuberculosis, % | 3.26 | 0.93 | 1.13 |  | 0.92 | 1.15 | 1.31 |
| History of spontaneous abortion, % | 0.00 | 0.58 | 9.92 |  | 0.00 | 12.80 | 16.72 |
| History of induced abortion, % | 0.00 | 1.33 | 57.63 |  | 0.00 | 79.51 | 91.27 |
| History of stillbirth, % | 0.00 | 0.36 | 6.22 |  | 0.00 | 7.69 | 10.85 |
| Number of live births, mean (SD) | - | 0.98 (0.15) | 2.36 (1.34) |  | 2.53 (1.40) | 2.10 (1.28) | 2.00 (1.26) |

a. Missing value = 47; b. Missing value = 3

BMI, body mass index; MET, metabolic equivalent of tasks

| Supplementary Table 2. Adjusted odds ratios (95% confidence intervals) for COPD risk associated with number of pregnancy losses, stratified by baseline characteristics | | | | | | | | | | | | |
| --- | --- | --- | --- | --- | --- | --- | --- | --- | --- | --- | --- | --- |
|  | **Number of participants** | **Number of events** | **Spontaneous abortion**  **OR (95% CI)** | ***p* for interaction** | **Number of participants** | **Number of events** | **Induced abortion**  **OR (95% CI)** | ***p* for interaction** | **Number of participants** | **Number of events** | **Stillbirth**  **OR (95% CI)** | ***p* for interaction** |
| **Age** |  |  |  | 0.81 |  |  |  | 0.12 |  |  |  | 0.77 |
| 30–39.9 | 47399 | 32 | 1.15 (0.80, 1.66) |  | 47399 | 281 | 1.34 (1.08, 1.65)** |  | 47399 | 11 | 1.27 (0.69, 2.33) |  |
| 40-49.9 | 92726 | 106 | 1.34 (1.09, 1.64)** |  | 92726 | 830 | 1.19 (1.05, 1.35)** |  | 92726 | 43 | 1.15 (0.85, 1.57) |  |
| 50-59.9 | 93118 | 235 | 1.23 (1.07, 1.41)** |  | 93118 | 1243 | 1.13 (1.03, 1.24)* |  | 93118 | 128 | 1.05 (0.87, 1.26) |  |
| ≥ 60 | 66339 | 397 | 1.14 (1.02, 1.28)* |  | 66339 | 1214 | 1.19 (1.09, 1.29)** |  | 66339 | 361 | 1.14 (1.02, 1.29)* |  |
| **BMI** |  |  |  | 0.20 |  |  |  | 0.89 |  |  |  | 0.97 |
| < 25 | 196774 | 505 | 1.15 (1.04, 1.26)** |  | 196774 | 2281 | 1.18 (1.10, 1.26)** |  | 196774 | 386 | 1.08 (0.97, 1.21) |  |
| ≥ 25 | 102808 | 265 | 1.30 (1.14, 1.48)** |  | 102808 | 1287 | 1.19 (1.08, 1.30)** |  | 102808 | 157 | 1.14 (0.96, 1.35) |  |
| **Region** |  |  |  | 0.34 |  |  |  | 0.43 |  |  |  | 0.73 |
| Rural | 166530 | 470 | 1.11 (0.999, 1.22) |  | 166530 | 1330 | 1.23 (1.14, 1.32)** |  | 166530 | 345 | 1.03 (0.92, 1.16) |  |
| Urban | 133052 | 300 | 1.31 (1.16, 1.48)** |  | 133052 | 2238 | 1.18 (1.09, 1.27)** |  | 133052 | 198 | 1.16 (0.99, 1.34) |  |
| **Educational level** |  |  |  | 0.97 |  |  |  | 0.18 |  |  |  | 0.22 |
| Elementary school and below | 170305 | 595 | 1.19 (1.09, 1.30)** |  | 170305 | 2008 | 1.16 (1.09, 1.24)** |  | 170305 | 477 | 1.14 (1.03, 1.26)** |  |
| Middle and high school | 116249 | 155 | 1.22 (1.03, 1.44)* |  | 116249 | 1348 | 1.25 (1.12, 1.38)** |  | 116249 | 57 | 0.88 (0.68, 1.16) |  |
| University and above | 13028 | 20 | 1.01 (0.63, 1.61) |  | 13028 | 212 | 1.09 (0.83, 1.42) |  | 13028 | 9 | 1.11 (0.55, 2.21) |  |
| **Household income** |  |  |  | 0.95 |  |  |  | 0.43 |  |  |  | 0.12 |
| < 5000 | 30367 | 155 | 1.09 (0.92, 1.31) |  | 30367 | 386 | 1.29 (1.12, 1.48)** |  | 30367 | 103 | 1.01 (0.82, 1.26) |  |
| 5000–19,999 | 147173 | 388 | 1.23 (1.10, 1.37)** |  | 147173 | 1469 | 1.19 (1.09, 1.28)** |  | 147173 | 261 | 1.09 (0.96, 1.25) |  |
| ≥ 20,000 | 12204 | 227 | 1.18 (1.02, 1.35)* |  | 12204 | 1713 | 1.17 (1.07, 1.27)** |  | 12204 | 179 | 1.18 (1.01, 1.38)* |  |
| **MET** |  |  |  | 0.14 |  |  |  | 0.10 |  |  |  | 0.42 |
| < 20 | 175745 | 563 | 1.21 (1.11, 1.33)** |  | 175745 | 2351 | 1.16 (1.08, 1.23)** |  | 175745 | 434 | 1.09 (0.98, 1.21) |  |
| 20–29.9 | 57617 | 99 | 1.004 (0.81, 1.24) |  | 57617 | 590 | 1.16 (1.02, 1.33)* |  | 57617 | 64 | 1.02 (0.79, 1.33) |  |
| ≥ 30 | 66220 | 108 | 1.27 (1.03, 1.55)* |  | 66220 | 627 | 1.27 (1.12, 1.46)** |  | 66220 | 45 | 0.94 (0.69, 1.27) |  |
| **Smoking status** |  |  |  | 0.24 |  |  |  | 0.06 |  |  |  | 0.98 |
| Never smoker | 284454 | 688 | 1.23 (1.14, 1.34)** |  | 284454 | 3236 | 1.18 (1.12, 1.25)** |  | 284454 | 484 | 1.11 (1.01, 1.22)* |  |
| Ever smoker | 15128 | 82 | 0.95 (0.75, 1.21) |  | 15128 | 332 | 1.20 (1.01, 1.42)* |  | 15128 | 59 | 1.03 (0.78, 1.37) |  |
| **Alcohol consumption** |  |  |  | 0.74 |  |  |  | 0.46 |  |  |  | 0.15 |
| Never drinker | 190625 | 551 | 1.20 (1.10, 1.32)** |  | 190625 | 2356 | 1.21 (1.13, 1.29)** |  | 190625 | 453 | 1.13 (1.02, 1.25)* |  |
| Ever drinker | 108957 | 219 | 1.18 (1.02, 1.36)* |  | 108957 | 1212 | 1.13 (1.03, 1.25)* |  | 108957 | 90 | 0.94 (0.75, 1.16) |  |
| **Tuberculosis** |  |  |  | 0.54 |  |  |  | 0.09 |  |  |  | 0.23 |
| No | 296254 | 742 | 1.20 (1.11, 1.30)** |  | 296254 | 3441 | 1.19 (1.13, 1.25)** |  | 296254 | 518 | 1.08 (0.98, 1.19) |  |
| Yes | 3328 | 28 | 1.04 (0.68, 1.59) |  | 3328 | 127 | 1.03 (0.77, 1.40) |  | 3328 | 25 | 1.36 (0.86, 2.15) |  |

Adjusted for age, region, BMI, level of highest education, annual household income, physical activity, smoking, and alcohol consumption. Analyses for pregnancy loss, spontaneous abortion, induced abortion, and stillbirth, were additionally adjusted for number of live births, and (where appropriate) number of spontaneous abortions, induced abortions, and stillbirths. Analyses for pregnancy, pregnancy loss, spontaneous abortion, induced abortion, and stillbirth, respectively, are among women with at least one pregnancy or at least one pregnancy loss, spontaneous abortion, induced abortion, stillbirth
BMI, body mass index; MET, metabolic equivalent of tasks
**p*<0.05; ***p*<0.01
